# Diagnosis of leukemic lung infiltration mimicking fungal infection by transbronchial lung cryobiopsy: the first case report

**DOI:** 10.1186/s12890-024-03300-6

**Published:** 2024-10-08

**Authors:** Duk Ki Kim, Chaeuk Chung, Dongil Park

**Affiliations:** 1 Division of Pulmonary and Critical Care Medicine, Department of Internal Medicine, Chungnam National University Hospital, 282 Munhwa-ro, Jung-gu, Daejeon, 35015 Republic of Korea; 2 Department of Internal Medicine, College of Medicine, Chungnam National University, 266 Munhwa-ro, Jung-gu, Daejeon, 35015 Republic of Korea

**Keywords:** Leukemia, Transbronchial lung cryobiopsy, Leukemic lung involvement, Radial EBUS, Cryotechnology

## Abstract

**Background:**

We here report the first case of leukemic lung infiltration diagnosed by transbronchial lung cryobiopsy (TBLC). TBLC is likely to be a superior method to transbronchial forceps biopsy because TBLC can get larger specimens, resulting in a higher chance of containing the leukemic cells infiltrated tissues. TBLC is generally considered a superior diagnostic method compared to transbronchial lung forceps biopsy (TBLB) because it utilizes cryotechnology to obtain larger specimens, increasing the likelihood of capturing tissues infiltrated with leukemic cells.

**Case presentation:**

A 69-year-old male patient with acute myeloid leukemia presented with a fever. His initial chest CT scans revealed consolidative lesions, raising suspicion of fungal infection such as angioinvasive aspergillosis or mucormycosis. TBLC and TBLB were conducted to achieve a precise diagnosis, and eventually, leukemic lung infiltration was identified exclusively in the tissues obtained from TBLC. Two cycles of chemotherapy was administrated to patient, showing improvements in symptoms and chest CT findings.

**Conclusions:**

TBLC has greater potential as a differential diagnostic method for pulmonary lesions than TBLB in leukemia patients facing therapeutic challenges due to its higher diagnostic yield.

## Background

Acute myeloid leukemia (AML) is a heterogenous neoplastic disorder characterized by uncontrolled proliferation of immature malignant myeloid cells and bone marrow failure, which is typically accompanied by a diverse range of complications including infection, anemia, bleeding, organ damage, and secondary cancer [1, 2]. Leukemic lung infiltration is defined as the accumulation of leukemic cells in extravascular space within the lung parenchyma in the absence of any other discernible etiology, such as infection, hemorrhage or hydrostatic congestion [2]. While it is most commonly observed in the terminal stage of AML, it can also occur at any stages. Autopsy studies have revealed that leukemic lung infiltration occurs in over 25% of patients with leukemia [3, 4]. However, in real clinical practice, it is difficult to diagnose leukemic lung infiltration antemortem due to the lack of characteristic radiologic or laboratory findings [5]. In recent years, the emergence of transbronchial lung cryobiopsy (TBLC) has led to the acquisition of larger specimens than previously possible, resulting in improved accuracy for diagnosing a wide range of medical conditions [6, 7]. Herein, we present a case of leukemic lung infiltration diagnosed by TBLC, which was not detected by conventional transbronchial lung biopsy (TBLB). To the best of our knowledge, this is the first report of leukemic lung infiltration diagnosed using TBLC.

## Case presentation

**Fig. 1:**
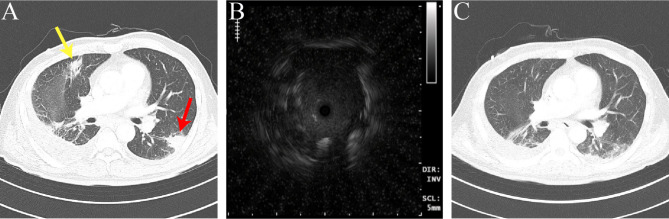
(**A**) An initial transverse CT scan reveals the presence of airspace consolidations measuring 2.1 and 2.2 cm-sized in the right upper lobe(yellow arrow) and left lower lobe(red arrow), respectively. A biopsy was performed on the consolidative lesion in the left lower lobe(red arrow). (**B**) Radial probe endobronchial ultrasound (RP-EBUS) image showing a concentric oriented lesion (‘within, concentric’ orientation) in consolidative lesion of left lower lobe. (**C**) Follow up CT scan after 2 cycles of Decitabine with Venetoclax demonstrates the disappearance of previous consolidative lesion

A 69-year-old male patient, who was diagnosed with AML M2 subtype with trisomy 8, presented with a fever of 38.3 degrees. The patient had undergone chemotherapy with Decitabine and Venetoclax, and the latest bone marrow biopsy, conducted three months ago, confirmed a morphological complete remission (CR) status of the disease. He has had type 2 diabetes mellitus and hypertension for 10 years, managed with oral medications. Moreover, there is no history of other lung diseases. The patient’s peripheral whole blood cell count showed 4140 cells/µl with the breakdown as follows: Seg. Neutrophil 40%, Lymphocyte 35%, Monocyte 6%, Eosinophil 3%, Myelocyte 2%, Immature Cell 14%, Nucleated red blood cell(NRBC) 3%. Additionally, C-reactive protein (CRP) and procalcitonin levels were elevated as 11.2 and 0.19, respectively, which may suggest an ongoing infection. Piperacillin/Tazobactam was empirically administered; however the fever persisted above 38 degrees without any signs of improvement. Therefore, a chest CT scan was performed to evaluate the presence of pulmonary infection, revealing newly developed airspace consolidations with inner cystic/necrotic changes in anterior segment of right upper lobe and superior segment of left lower lobe (Fig. 1A). Radiologic interpretation indicated the possibility of a fungal infection such as angioinvasive aspergillosis or mucormycosis. Sputum fungus stain/AFB stain/TB-PCR tests showed negative results, and both sputum culture and blood culture were also negative. To evaluate for the consolidative lesions seen on the chest CT scan, endobronchial ultrasound-guided transbronchial lung biopsy with a guide sheath (EBUS-GS-TBLB) was performed using 1.5 mm forceps (FB-233D, Olympus Medical, Japan). Briefly, after administering conscious sedation using propofol and midazolam, 13 mm ballon catheter (B7-2 C; Olympus, Japan) was positioned at the enrance of the left lower lobe bronchus. Subsequently in_tubation was performed using 7.5 Fr endotracheal tube. Bronchoscopy was then conducted through the endotracheal tube, and a radial probe endobronchial ultrasound (RP-EBUS) was performed to localize the target (Fig. 1B). Cryobiopsy was then performed through a guide sheath using a 1.1 mm cryoprobe (Erbe Elektromedizin GmbH, Germany), with immediate ballooning after en-bloc removal with cryoprobe to prevent the spread of bleeding. Moderate bleeding after the cryobiopsy was well-managed using bronchoscopic suction without infusion of vasoactive agents or cold saline. After the biopsy, bronchoalveolar lavage (BAL) was performed, followed by AFB stain, Fungal stain, Gram stain, AFB culture, Fungal culture, Bacterial culture, TB PCR, and NTM PCR. No specific findings were identified.

**Fig. 2:**
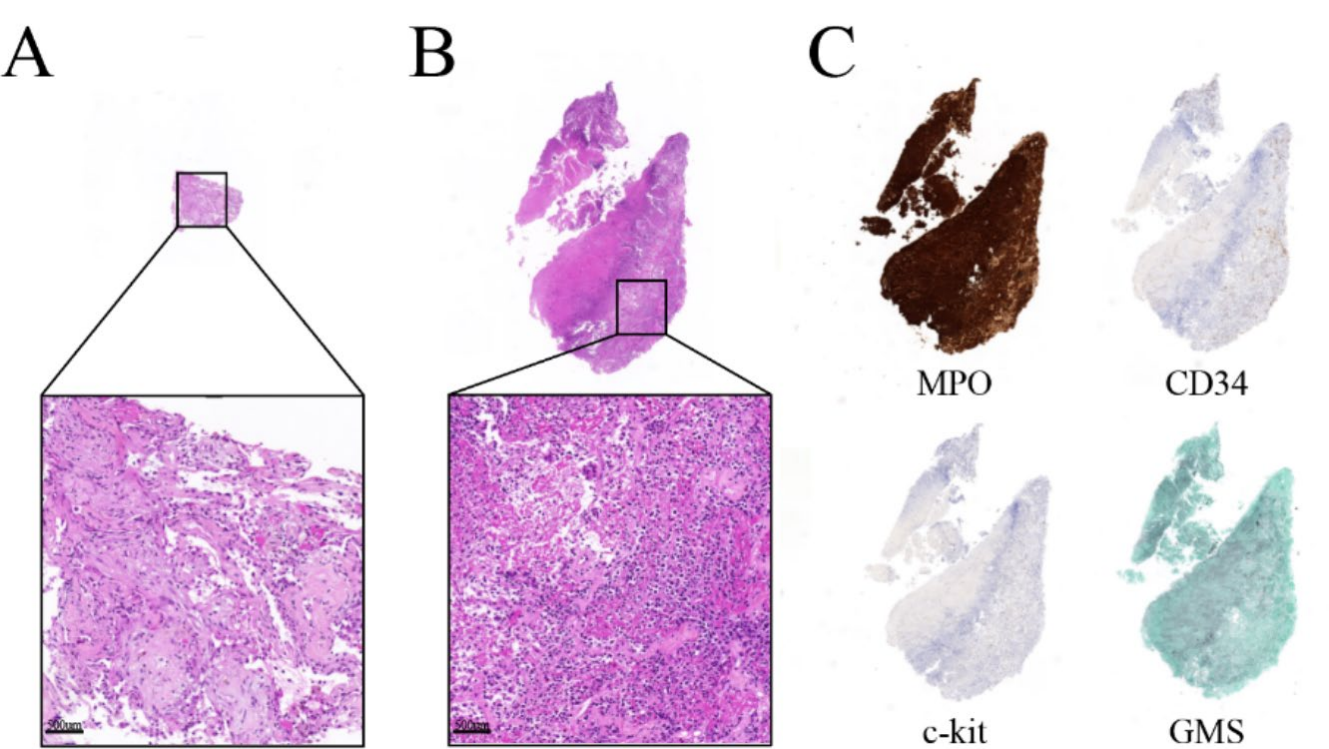
Pathologic findings of (**A**) TBLB(H&E) and (**B**) TBLC(H&E). Unlike TBLB, inflammatory soft tissue with necrosis was observed in TBLC. (**C**) Additional immunohistochemical staining using MPO, CD34, c-kit and GMS revealed that the consolidative lesion on chest CT scan was indicative of leukemic lung infiltration. H&E: Hematoxylin and Eosin

The specimen of the TBLB only showed non-neoplastic lung parenchyma (Fig. 2A). On the other hand, the result of the TBLC showed acute and chronic inflammation with necrosis and atypical cells (Fig. 2B). Additional immunohistochemical staining revealed pulmonary leukemic infiltration based on positive findings of Myeloperoxidase(MPO) staining (Fig. 2C) and negative CD34/c-kit/Grocott’s methenamine silver(GMS) staining (Fig. 2C). After the diagnosis of the leukemic lung infiltration, the patient received 2 cycles of Decitabine with Venetoclax, and follow-up Chest CT showed improvement in the consolidations (Fig. 1C).

## Discussion and conclusions

AML comprises approximately 80% of acute leukemia cases and is characterized by aberrant proliferation and differentiation status of myeloid stem cells, resulting in diverse complications associated with the disease itself or treatment toxicity [8]. Notably, patients with AML frequently experience wide range of life-threatening pulmonary complications including bacterial pneumonia, fungal infection, hemorrhage, edema and leukemic lung involvement which lead to mortality rates reaching up to 50%. Unlike other complications, leukemic lung involvement is a distinct complication directly related to leukemic cells and manifests as follows: (i) leukemic infiltration, accumulation of blasts into the extravascular space of lung parenchyma, (ii) leukostasis, the emergent medical condition that results from the intravascular accumulation of leukemic cells resulting in pulmonary edema and respiratory distress, (iii) acute leukemic cell lysis pneumopathy, an acute respiratory distress syndrome caused by toxic and thromboplastic substances caused by rapid destruction of leukemic cells after chemotherapy [2].

The presence of leukemic lung infiltration has been identified through autopsy in approximately 20–30% of AML cases [3]. However, diagnosing leukemic lung infiltration is often challenging due to the absence of specific clinical manifestations or radiological findings that are distinct from other common pulmonary complications such as infections or hemorrhage [9]. Moreover, the diagnostic procedure using conventional TBLB and/or BAL procedures may be limited, with a diagnostic yield typically less than 50% [10, 11].

TBLC is a novel bronchoscopic technique compared with conventional transbronchial forceps biopsies to obtain larger tissue samples from patients suspected of lung cancer, interstitial lung disease, or lung rejection after transplantation. TBLC uses cryotechnology based on the Joule-Thomson effect, in which compressed gas is released at high flow rate, rapidly creating a very low temperature. This freezing process induces the adhesion of tissue to the probe, allowing for subsequent *en-bloc* removal [12]. TBLC allows obtaining tissue approximately 26 times larger compared to TBLB, and it is also possible to obtain a sufficiently large artifact-free tissue area upon examination, contributing to a higher diagnostic yield [13]. Recent studies indicate that incorporating cryobiopsy into conventional procedures for sampling peripheral pulmonary lesions enhances diagnostic yield to approximately 90–94% [14, 15]. This improvement is primarily due to the increased accessibility of areas adjacent to the lesions. Furthermore, cryobiopsy destroys benign tissue, allowing for the removal of normal structures, such as bronchial walls, situated between the radial endobronchial ultrasound (R-EBUS) probe and the target lesion in the ‘adjacent to’ orientation. This increase in diagnostic yield occurs because the cryoprobe can be positioned adjacent to the target lesion, resulting in a higher yield compared to positioning it within the lesion [12]. In this case, leukemic lung infiltration that was not diagnosed by TBLB could be diagnosed with TBLC, leading to successful management. Reported pathologic findings of leukemic lung infiltration include the presence of leukemic cells in the extravascular space along the peribronchial and perivascular regions [16]. Therefore, in cases of leukemic lung infiltration, the lesions are typically located within normal structures such as the bronchus or vascular regions. Consequently, cryobiopsy, which allows for en-bloc removal and thereby eliminates the barriers posed by normal tissues, is considered to offer a diagnostic advantage over transbronchial lung biopsy (TBLB) for diagnosing leukemic lung infiltration.

In conclusion, we report the first case of leukemic lung infiltration diagnosed using TBLC. The generalizability of the diagnostic method depicted in this report is limited due to the absence of other documented studies. Nonetheless, this case exemplified the potential efficacy of TBLC in the differential diagnosis of pulmonary lesions in leukemia patients encountering therapeutic challenges.

## Data Availability

The images incorporated in this article have been included as figures. The raw materials are not available in adherence to patient privacy concerns.

## Abbreviations

TBLB: Transbronchial Lung Biopsy
TBLC: Transbronchial Lung Cryobiopsy
AML: Acute myeloid leukemia
CR: Complete remission
NRBC: Nucleated red blood cell
CRP: C-reactive protein
EBUS-GS-TBLB: Endobronchial ultrasound-guided transbronchial lung biopsy with a guide sheath
RP-EBUS: Radial probe endobronchial ultrasound
MPO: Myeloperoxidase
GMS: Grocott’s methenamine silver
BAL: Bronchoalveolar lavage
